# Multi-volume hemacytometer

**DOI:** 10.1038/s41598-021-93477-1

**Published:** 2021-07-08

**Authors:** Ravangnam Thunyaporn, Il Doh, Dong Woo Lee

**Affiliations:** 1 Department of Biomedical Engineering, Konyang University, Daejeon, 35365 Republic of Korea; 2 Korea Research Institute of Standards and Science, Daejeon, Korea; 3 Central R & D Center, Medical & Bio Decision (MBD) Co., Ltd, Suwon, Republic of Korea

**Keywords:** Biological techniques, Biotechnology, Engineering

## Abstract

Cell counting has become an essential method for monitoring the viability and proliferation of cells. A hemacytometer is the standard device used to measure cell numbers in most laboratories which are typically automated to increase throughput. The principle of both manual and automated hemacytometers is to calculate cell numbers with a fixed volume within a set measurement range (10^5^ ~ 10^6^ cells/ml). If the cell concentration of the unknown sample is outside the range of the hemacytometer, the sample must be prepared again by increasing or decreasing the cell concentration. We have developed a new hemacytometer that has a multi-volume chamber with 4 different depths containing different volumes (0.1, 0.2, 0.4, 0.8 µl respectively). A multi-volume hemacytometer can measure cell concentration with a maximum of 10^6^ cells/ml to a minimum of 5 × 10^3^ cells/ml. Compared to a typical hemacytometer with a fixed volume of 0.1 µl, the minimum measurable cell concentration of 5 × 10^3^ cells/ml on the multi-volume hemacytometer is twenty times lower. Additionally, the Multi-Volume Cell Counting model (cell concentration calculation with the slope value of cell number in multi-chambers) showed a wide measurement range (5 × 10^3^ ~ 1 × 10^6^ cells/ml) while reducing total cell counting numbers by 62.5% compared to a large volume (0.8 µl-chamber) hemacytometer.

## Introduction

Cell counting is a method used to determine the concentration of cells in a sample volume. For example, in cell culture experiments counting cells is vital to monitor cell viability, proliferation rate, immortalization or transformation, seeding, or preparing cells in subsequent experiments, transfection, or infection^1^. Diagnostically, the concentration of white blood cells and red blood cells can assist those in the medical field to identify specific diseases and can predict severity of disease states such as coronary atherosclerosis^2–4^, HIV detection from whole-blood^5, 6^, etc.

The hemacytometer was first invented over a century ago to count blood cells. Presently the hemacytometer remains the gold standard for cell counting and is used by most laboratories to count cells as it is both inexpensive and versatile^7^. A hemacytometer is made from optical glass for use under a microscope and consists of 2 parts: a thick glass slide and cover glass with a small gap to contain cell suspension in a 0.1 µl small grid area size 1 × 1 mm^8^. It cannot be accurately measured in high concentrations because the cells too crowned and difficult to count. The cells have to be diluted and counting again^9^.

Over the last two decades, automated hemacytometers have been developed to increase the throughput of counting a large number of samples in a short period of time. Automation also reduces human error in the cell counting process and automatic hemacytometers are commonly used in laboratories for these reasons. In general, hemacytometers are composed of a digital camera and a slide or cartridge containing the cell suspension to be counted. The digital camera captures cells on the slide in the fixed volume and analyzes them using specialized software for cell counters to calculate cell concentration based on the fixed volume^6^. Both manual and automated hemacytometers use a fixed volume to measure cell concentration. Though manual and automated hemacytometers are standard in most laboratories, they cannot measure low cell concentration samples and have a narrow measurement range (10^5^ ~ 10^6^ cells/ml) due to the fixed volume containing cell. Most hemacytometers count cells in 0.1 µl fixed volume where one cell exists at 10^4^ cells/ml sample. The range of hemacytometer is approximately 10^5^ cells/ml. For example, the Cedex HiRes Analyzer which is an automatic hemacytometer, can measure cell density ranging from 3.13 × 10^5^ to approximately 1.0 × 10^7^ cells/ml^10^. The optimal range of Cellometer cell counters is approximately 1 × 10^5^ to 1 × 10^6^ cells/ml^11^.

The previous study has shown that the size of the fixed control volume of cells in the suspension increases, the number of cells is also increased which can be extended to measure cells in a wide range of concentrations^12, 13^. The limit of the range of cell concentration measurement is due to the fixed volume of the sample which causes the minimum and maximum measurable cell concentration to decrease or increase. A typical 0.1 µl fixed volume (most used volume in hemacytometers) contains 10 ~ 500 cells at 10^5^ ~ 5 × 10^6^ cells/ml while a 0.2 µl fixed volume contains 10 ~ 500 cells at 5 × 10^4^ ~ 2.5 × 10^6^ cells/ml. Therefore, if a sample of unknown concentration is out of range of the hemacytometer, the sample must be prepared again by increasing or decreasing the cell concentration.

**Figure 1:**
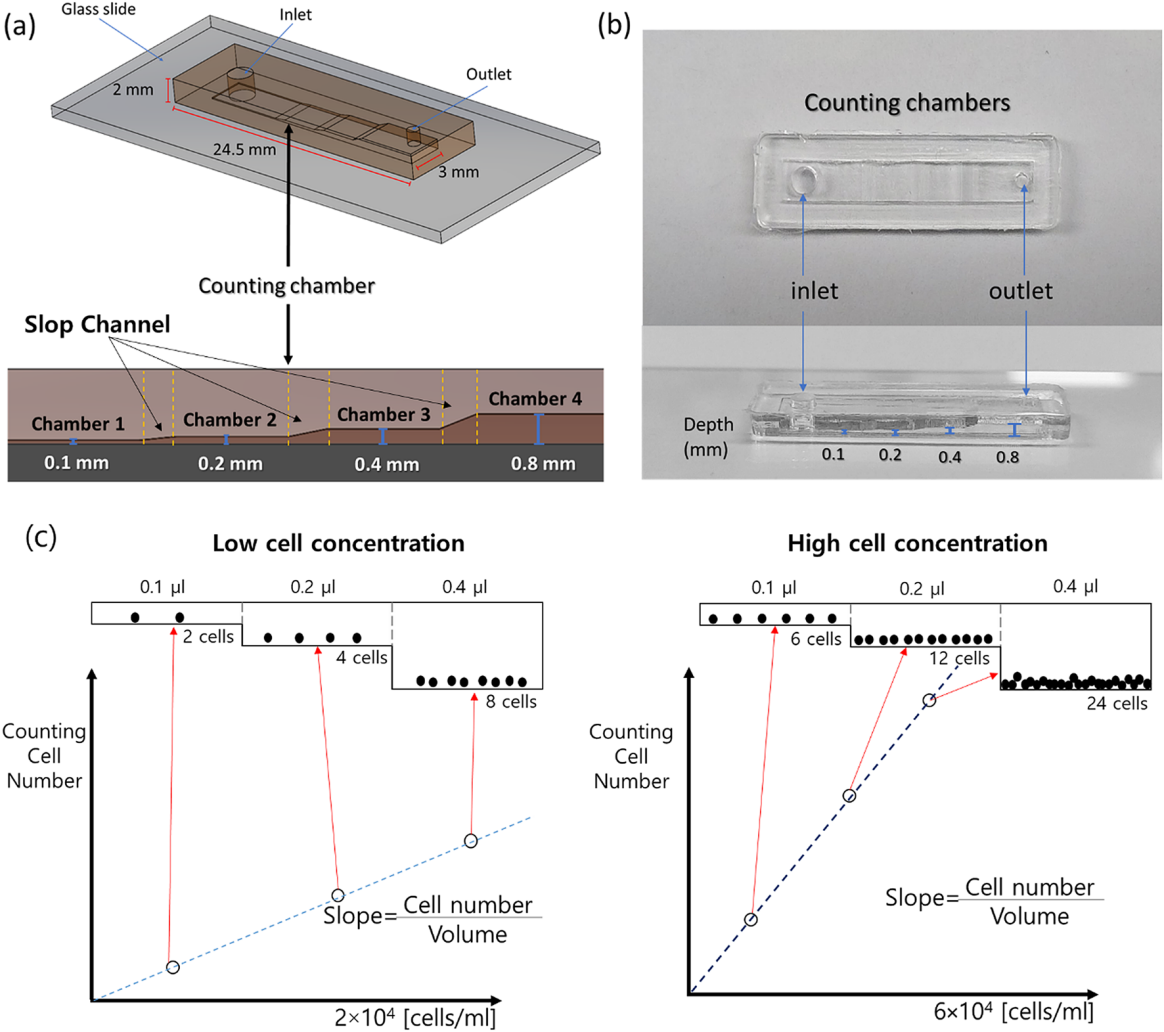
Schematic view of the multi-volume hemacytometer. (**a**) The counting chamber was attached to a glass slide that had an inlet for pipetting and an outlet for airflow. The cell counting chamber consisted of four chambers of varying depths including 0.1, 0.2, 0.4, 0.8 mm respectively. (**b**) The PDMS fabrication device. (**c**) Counting cell number in each chamber are proportional to the volume of the chamber when a sample flowed in the chambers having different volumes. In case of low cell concentration sample, slope which mean cells per unit volume is small. However, in high cell concentration sample, the slop is large due to large different of counting cells per chamber.

To solve this problem, the current study proposed the use of a multi-volume hemacytometer. This device counts cells in four chambers at volumes of 0.1, 0.2, 0.4, 0.8 µl as shown in Fig. 1. A multi-volume cell counter can measure cell concentrations ranging from 10^6^ cells/ml to 5 × 10^3^ cells/ml. Compared to a hemacytometer having a fixed volume of 0.1 µl, the minimum measurable cell concentration is 5 × 10^3^ cells/ml which is twenty times lower. Additionally, the Multi-Volume Cell Counting model (cell concentration calculation with the slope value of the cell number in multi-chambers) was also validated to measured cell concentration and increases the measurement speed as a sample of only 1.5 µl can measure the wide range of cell concentrations.

## Results

To compare the accuracy and range of cell concentration measurement between the conventional hemacytometer and the proposed multi-volume hemacytometer, we measured five samples at 10^3^, 5 × 10^3^, 10^4^, 10^5^, and 10^6^ cells/ml. In the case of the multi-volume hemacytometer, there are two cell concentration calculation models necessary to determine the cell concentration. In the conventional Fixed-Volume Cell Counting (FVCC), the cell concentrations were calculated by counting cells in each chamber at different volumes. In the Multi-Volume Cell Counting (MVCC) model, the cell concentrations were calculated using the slope (cell number growth per volume) in the graph of cell number by volume as show in Fig. 1c.

### Fixed-volume cell counting (FVCC) model

**Figure 2:**
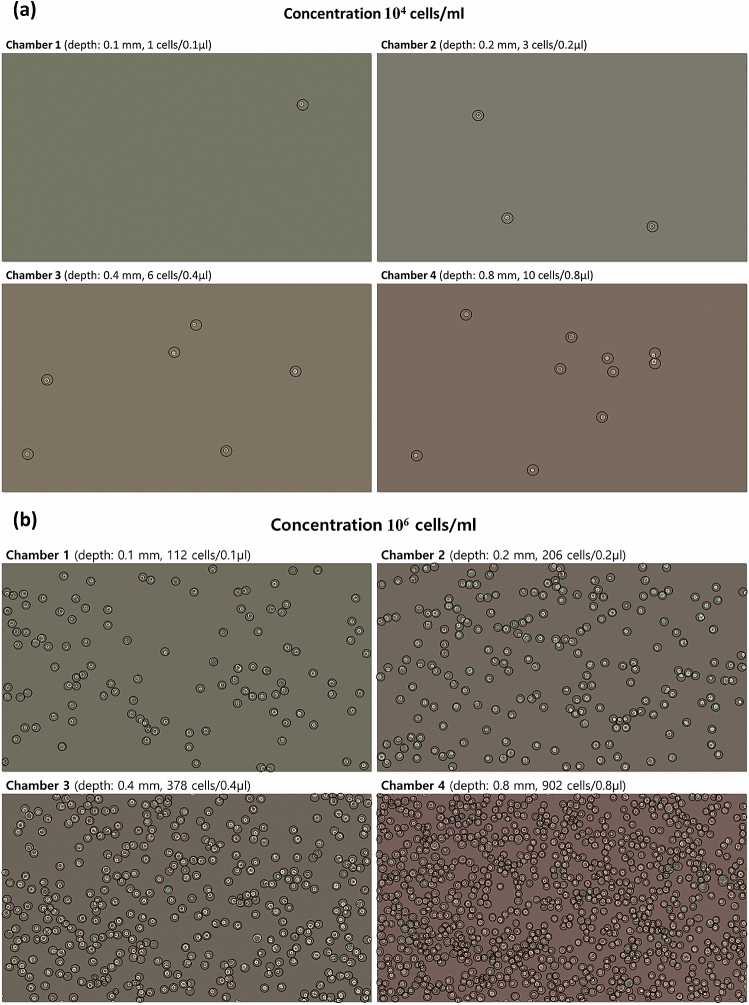
Microscopic cell images in multiple volumes (0.1, 0.2, 0.4, and 0.8 µl) in (**a**) low concentration at 10^4^ cells/ml, and (**b**) high concentration at 10^6^ cells/ml liquid sample (under 40 × magnification).

A hemacytometer has a ruled surface below the cover glass of 0.1 mm which limits the volume of liquid suspension to 0.1 µl per one of nine squares and restricts it to accurately measure the concentration of cells in the range of 2 × 10^5^ ~ 2.5 × 10^6^ cells/ml^9^. As shown in Table 1, in the current experiment the hemacytometer shows a large standard deviation (more than 15%) in the samples lower than 10^5^ cells/ml. The proposed device has several chambers with different volumes such as 0.1, 0.2, 0.4, and 0.8 µl to contain the multi-volume of cells in suspension. Figure 2 shows that the number of cells varied in conjunction with the volume. The number of cells increased with the volume of the chamber. As shown in Table 1, chamber 1, 2, 3, 4, and the MVCC model were used to measure cell concentration. In the current experiments, chamber 1 in area 1 mm^2^ contained a volume of 0.1 µl equal to the volume of the standard hemacytometer. In the low volume chamber, the number of cells was not great enough to calculate the cell concentration. In contrast, as the volume of the chamber increased, the cells also increased enough to allow the cell concentration to be calculated. Therefore, chamber 4 has a wide measurement range and was able to be measured at the lowest concentration at 5 × 10^3^ cells/ml. However, in the maximum cell concentration of 10^6^ cells/ml, it was difficult to count a high number of cells in a large volume (0.8 µl) and repeat the measurement five times. Therefore, the user must select the appropriate chamber volume according to the sample.

**Table 1:** Comparison of cell concentration measurements between a standard hemacytometer and a multi-volume hemacytometer.

Sample	Concentration unit	Hemacytometer (volume: 0.5 µl)	Multi-volume hemacytometer				
			FVCC Model*				MVCC model ** (volume: 1.5 µl)
			Chamber 1 (volume: 0.5 µl)	Chamber 2 (volume: 1 µl)	Chamber 3 (volume: 2 µl)	Chamber 4 (volume: 4 µl)	
		Mean ± SD (%RSD)	Mean ± SD (%RSD)	Mean ± SD (%RSD)	Mean ± SD (%RSD)	Mean ± SD (%RSD)	Fit values ± SD (%RSD)
1	10^6^ cells/ml	1.08 ± 0.11 (9.7%)	1.14 ± 0.02 (1.6%)	0.99 ± 0.07 (7.0%)	1.14 ± 0.07 (5.7%)	1.02 ± 0.03 (3.3%)	1.04 ± 0.03 (2.5%)
2		1.18 ± 0.13 (10.8%)	1.14 ± 0.11 (9.3%)	0.94 ± 0.06 (6.7%)	1.08 ± 0.10 (9.6%)	1.14 ± 0.05 (4.5%)	1.12 ± 0.04 (3.6%)
3		1.05 ± 0.09 (8.5%)	1.07 ± 0.16 (14.9%)	1.09 ± 0.15 (17%)	1.08 ± 0.16 (14.4%)	1.07 ± 0.01 (1.3%)	1.07 ± 0.03 (3.1%)
4		1.16 ± 0.12 (10.5%)	0.88 ± 0.06 (6.5%)	0.94 ± 0.04 (4.6%)	1.08 ± 0.15 (14.0%)	1.12 ± 0.07 (6.5%)	1.10 ± 0.03 (3.0%)
5		1.19 ± 0.14 (11.3%)	1.12 ± 0.17 (15.2%)	0.92 ± 0.06 (6.8%)	0.94 ± 0.07 (7.2%)	1.05 ± 0.13 (13.3%)	1.03 ± 0.11 (10.4%)
	AVG	**1.13 ± 0.06**	**1.07 ± 0.11**	**0.98 ± 0.08**	**1.07 ± 0.08**	**1.08 ± 0.06**	**1.07 ± 0.04**
	%RSD	**5.61**	**10.51**	**6.89**	**6.86**	**4.54**	**3.64**
1	10^5^ cells/ml	1.38 ± 0.35 (25.3%)	1.26 ± 0.27 (21.4%)	1.14 ± 0.20 (17.4%)	0.94 ± 0.12 (11.5%)	1.22 ± 0.10 (8.3%)	1.17 ± 0.02 (1.5%)
2		1.24 ± 0.42 (34.0%)	1.12 ± 0.34 (30.5%)	1.07 ± 0.15 (14.3%)	1.18 ± 0.30 (25.7%)	1.26 ± 0.08 (6.4%)	1.23 ± 0.09 (7.7%)
3		1.20 ± 0.43 (35.8%)	1.16 ± 0.49 (42.1%)	1.28 ± 0.47 (36.9%)	1.20 ± 0.27 (22.3%)	1.21 ± 0.11 (9.0%)	1.21 ± 0.11 (9.4%)
4		1.22 ± 0.35 (28.6%)	1.16 ± 0.43 (37.4%)	1.21 ± 0.19 (15.3%)	1.28 ± 0.29 (22.5%)	1.26 ± 0.12 (9.6%)	1.26 ± 0.13 (10.2%)
5		1.20 ± 0.53 (44.1%)	1.00 ± 0.16 (15.8%)	1.28 ± 0.27 (21.1%)	1.13 ± 0.23 (19.9%)	1.30 ± 0.10 (8.0%)	1.26 ± 0.10 (8.1%)
	AVG	**1.25 ± 0.08**	**1.14 ± 0.09**	**1.20 ± 0.09**	**1.15 ± 0.13**	**1.25 ± 0.04**	**1.23 ± 0.04**
	%RSD	**6.06**	**8.23**	**7.63**	**11.10**	**6.06**	**3.17**
1	10^4^ cells/ml	1.40 ± 1.14 (81.4%)	1.40 ± 1.14 (81.4%)	1.20 ± 0.57 (47.5%)	1.15 ± 0.14 (11.9%)	1.18 ± 0.14 (12.1%)	1.17 ± 0.10 (8.7%)
2		1.00 ± 0.71 (70.7%)	1.00 ± 1.00 (100%)	1.10 ± 0.65 (59.3%)	1.15 ± 0.42 (36.4%)	1.20 ± 0.19 (15.8%)	1.22 ± 0.12 (9.5%)
3		0.60 ± 0.55 (91.3%)	1.40 ± 0.89 (63.9%)	1.20 ± 0.57 (47.5%)	1.15 ± 0.38 (33.0%)	1.15 ± 0.27 (23.6%)	1.16 ± 0.22 (19.0%)
4		1.00 ± 0.71 (70.7%)	1.20 ± 0.83 (69.7%)	1.20 ± 0.57 (47.5%)	1.10 ± 0.29 (25.9%)	1.28 ± 0.29 (22.4%)	1.24 ± 0.18 (14.3%)
5		1.00 ± 0.71 (70.7%)	0.80 ± 0.45 (55.9%)	1.00 ± 0.50 (50.0%)	1.15 ± 0.52 (45.1%)	1.18 ± 0.59 (22.1%)	1.16 ± 0.23 (19.9%)
	AVG	1.00 ± 0.28	1.16 ± 0.26	**1.14 ± 0.09**	**1.14 ± 0.02**	**1.20 ± 0.05**	**1.19 ± 0.04**
	%RSD	28.28	22.48	**7.85**	**1.96**	**4.02**	**3.19**
1	5 × 10^3^ cells/ml	4.00 ± 5.58 (136.9%)	8.00 ± 4.47 (55.9%)	2.00 ± 2.74 (136.9%)	7.00 ± 3.26 (46.6%)	5.50 ± 1.43 (25.9%)	5.65 ± 1.27 (22.4%)
2		6.00 ± 5.48 (91.3%)	4.00 ± 8.94 (223.6%)	9.00 ± 9.62 (106.9%)	6.00 ± 2.85 (47.5%)	5.25 ± 2.85 (54.3%)	5.54 ± 3.06 (55.3%)
3		2.00 ± 4.47 (223.6%)	4.00 ± 8.94 (223.6%)	4.00 ± 4.18 (104.6%)	7.50 ± 2.50 (33.3%)	6.00 ± 1.63 (27.2%)	6.16 ± 1.17 (19.0%)
4		2.00 ± 4.47 (223.6%)	6.00 ± 8.94 (149.1%)	7.00 ± 8.37 (119.5%)	6.00 ± 1.37 (22.8%)	6.25 ± 1.77 (28.3%)	6.24 ± 1.67 (28.8%)
5		8.00 ± 8.37 (104.6%)	2.00 ± 4.47 (223.6%)	6.00 ± 2.24 (37.3%)	5.50 ± 2.09 (38.0%)	5.25 ± 2.05 (39.1%)	5.29 ± 1.90 (36.0%)
	AVG	4.40 ± 2.61	4.80 ± 2.28	5.60 ± 2.70	**6.40 ± 0.82**	**5.65 ± 0.45**	**5.78 ± 0.41**
	%RSD	59.27	47.51	48.25	**12.84**	**8.04**	**7.06**
1	10^3^ cells/ml	0	0	0	1.50 ± 1.37 (91.3%)	1.50 ± 0.56 (37.3%)	1.41 ± 0.33 (23.6%)
2		2.00 ± 4.47 (223.6%)	0	0	1.50 ± 1.37 (91.3%)	1.25 ± 0.88 (70.7%)	1.22 ± 0.85 (69.9%)
3		4.00 ± 5.48 (136.9%)	2.00 ± 4.47 (223.6%)	1.00 ± 2.24 (223.6%)	2.00 ± 1.77 (70.7%)	2.00 ± 0.68 (34.2%)	2.05 ± 0.83 (40.6%)
4		2.00 ± 4.47 (223.6%)	2.00 ± 4.47 (223.6%)	1.00 ± 2.24 (223.6%)	2.50 ± 3.26 (163%)	1.75 ± 1.43 (81.4%)	1.76 ± 0.70 (39.4%)
5		0	0	1.00 ± 2.24 (223.6%)	2.50 ± 3.06 (122%)	1.75 ± 2.43 (81.4%)	1.84 ± 0.89 (48.3%)
	AVG	1.60 ± 1.67	0.80 ± 1.10	0.60 ± 0.55	2.00 ± 0.50	1.65 ± 0.29	1.66 ± 0.33
	%RSD	104.58	136.93	91.28	25.00	17.28	20.10

*FVCC Model: Fixed-Volume Cell Counting Model.

**MVCC Model: Multi-Volume Cell Counting Model.

Bold values Cell concentration measurement quality control pass (%RSD < 15%).

### Multi-volume cell counting (MVCC) model

**Figure 3:**
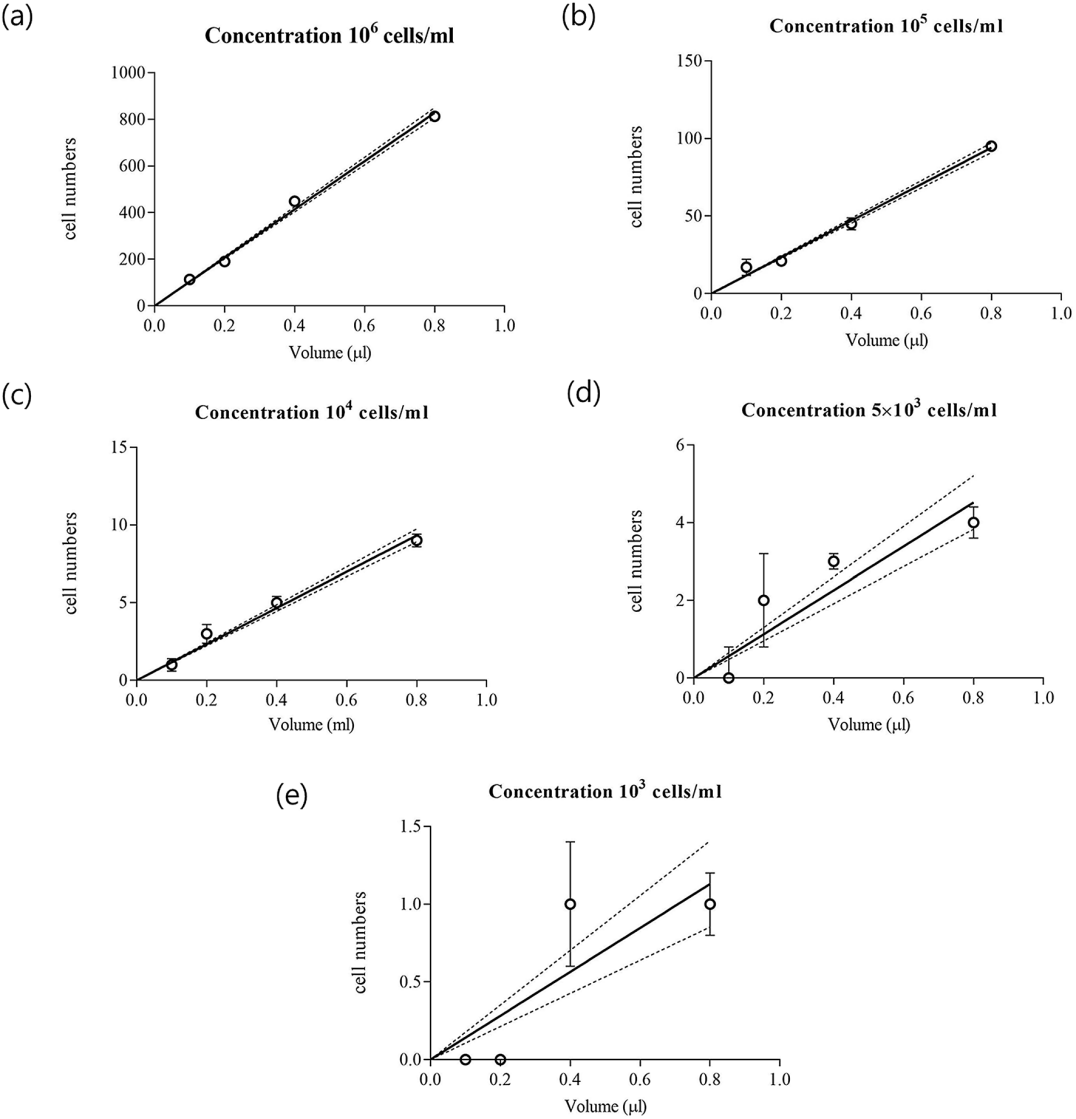
The cell numbers according to chamber volume in different cell concentration. (**a**) 10^6^ cells/ml, (**b**) 10^5^ cells/ml, (**c**) 10^4^ cells/ml, (**d**) 5 × 10^3^ cells/ml, and (**e**) 10^3^ cells/ml. Each point is the average of cell number and error bar is standard deviation in five replications. The slopes from the linear equations were calculated for measuring cell concentration.

**Figure 4:**
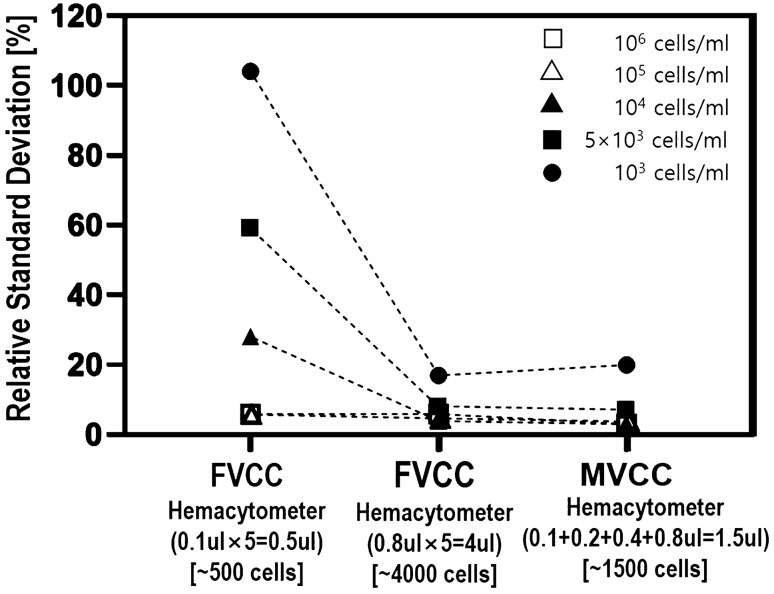
Comparison of the percent of relative standard deviation of cell number in each concentration between a standard hemacytometer and a multi-volume hemacytometer with four chambers and the MVCC model.

Figure 3 shows the cell number measurement by chamber volume and the slope in the Multi-Volume Cell Counting (MVCC) model. The slope indicates the cell number per volume, reflecting the cell concentration. The slope (cell numbers per volume) was calculated by the cell number in 0.1, 0.2, 0.4, and 0.8 µl chambers. Dotted lines indicate a fit for standard error. As the sample cell concentration decreased, the spacing between the dotted lines increased. In the repeated cell concentration measurement of 5 × 10^3^ cells/ml sample, the MVCC model indicates the standard deviation of cell concentration is lower than 15%, while the FVCC model indicates a higher standard deviation. As shown in Fig. 4, measurement using the conventional hemacytometer shows that when the sample concentration was low, the relative standard deviation of measuring cell concentration increased significantly. The measurement range of the conventional hemacytometer is 10^6^ ~ 10^5^ cells/ml, and using a 0.8 µl chamber showed a wide measurement range of 10^6^ ~ 5 × 10^3^ cells/ml. However, in 10^6^ cells/ml, the hemacytometer using a 0.8 µl chamber had too many cells to count manually (approximately 4000). Figure 4 shows how the proposed MVCC model reduced the number of cell counts needed to measure cell concentration without reducing the measurement range. The MVCC model measured 5 × 10^3^ ~ 10^6^ cells/ml within a 10% standard deviation and reduced the counting cell number by about 62.5%. The MVCC model reduced the counting burden in large volumes as a total volume of only 1.5 µl (0.1 + 0.2 + 0.4 + 0.8 µl) was needed to measure the cell concentration at a wide range of concentrations.

## Discussion

As well known, the hemacytometer is a basic device used to count cells in most laboratories. Many times a cell sample needs to be re-prepared by either increasing or decreasing the cell concentration because the unknown cell concentration is outside the range of the hemacytometer. Thus, our study proposed a novel device to measure cell concentration using a multi-volume hemacytometer. This device is designed to have a multi-chamber that can contain multi-volume such as 0.1, 0.2, 0.4, and 0.8 μl, expanding the contained volume for a hemacytometer. Compared to a standard hemacytometer with a fixed volume of 0.1 μl, the minimum measurable cell concentration for the multi-chamber hemacytometer is 5 × 10^3^ cells/ml, which is twenty times lower.

This is because the multi-chamber hemacytometer has 4 chambers that can hold different amounts of cell samples from low to high volume (0.1, 0.2, 0.4, and 0.8 µl). Cell concentration in each chamber can be calculated by the average number of cells in the area divided by the fixed volume. The number of cells increases as the chamber volume increases, i.e. at fixed volume 0.1 μl, there are 10 ~ 500 cells at 10^5^ ~ 5 × 10^6^ cells/ml while at fixed volumes 0.2 μl, there are 10 ~ 500 cells at 5 × 10^4^ ~ 2.5 × 10^6^ cells/ml. This multi-volume chamber expands the range of the cell concentration measurements compared to the standard hemacytometer. When unknown sample was loaded in multi-volume hemacytometer, the cell concentration can be measured by selecting an appropriate chamber with the number of cells (10 to 200 cells/chamber) out of four chambers.

In this study, phosphate buffered saline (PBS) is used to dilute the cell concentration. Therefore, the sample viscosity is similar to PBS, allowing the sample to flow easily into the chip. At low cell concentrations, high-volume chambers can provide a clearer accurate cell count cell on bottom than high cell concentrations. Therefore, at high cell concentrations in high volume chambers, we recommend changing the focus to verify that the cells are completely precipitated before taking images. Basically, we use a cancer cell line which concentrations 5 × 10^3^ ~ 10^6^ cells/ml in PBS. In the case of blood cells, RBC has a concentration of approximately 5 × 10^12^ cells/L (5 × 10^6^ cells/µl)^14^. This sample must be diluted to 10^4^ ~ 10^6^ cells/ml (1:10,000 dilution) with PBS before counting the cell by using this device.

In addition, the multi-volume hemacytometer proposed the Multi-Volume Cell Counting (MVCC) model for cell concentration measurement. Chamber 4 contains a maximum volume of 0.8 μl and has a wide measurement range that able to measure the lowest concentration at 5 × 10^3^ cells/ml. Nonetheless, if cell concentration was high around 1 × 10^6^ cells/ml, cell counting only in 0.8 μl-chamber was difficult and used a lot of time to determine the accuracy of the cell concentration. So, we proposed and validated the Multi-Volume Cell Counting (MVCC) model that cell concentration calculation with the slope value of cell numbers in multi-chambers. The MVCC showed a wide measurement range of 5 × 10^3^ ~ 1 × 10^6^ cells/ml while reducing total cell counting numbers by approximately 62.5% compared to a 0.8 μl-chamber hemacytometer.

## Materials and methods

### Multi-volume hemacytometer design

The multi-volume hemacytometer is designed to address the problem of loading cell samples in unknown concentrations which can fall outside the range of a standard hemacytometer. The proposed design defines multiple volumes as it consists of 4 chambers with different depths as follows: chamber 1 depth 0.1 mm, chamber 2 depth 0.2 mm, chamber 3 depth 0.4 mm, and chamber 4 depth 0.8 mm which are connected within the same sheet. Each chamber has an area of 3 × 3 mm^2^ and connected by slope channel. The slope channel has 3 mm wide 1 mm length. The height of slope channel increases gradually from the lower chamber height to the higher chamber height as shown in Fig. 1. We designed the slope channel to avoid air bubble trap when the chamber height suddenly increases. The air bubbles make the cells bad dispersion in chambers. The slope also helps the cells spread continuously from chamber to chamber without damaging the flow. The counter chamber and slop channel are made using the PDMS production process after which the PDMS sheets are bonded on a slide glass (Fig. 1).

### Multi-volume hemacytometer fabrication

This cell counter sheet is made using polydimethylsiloxane (PDMS) with s SYLGARD 184 silicone elastomer base and SYLGARD 184 silicone elastomer curing agent. The mold was made of aluminium by computer numerical control (CNC) machining. In the first step silicone elastomer base and silicone curing agent were mixed at a 10:1 ratio and a desiccator was used to burst any bubbles for 15 min before the elastomer mix was poured into a cell counter aluminium mold and placed inside a desiccator for 15 min. The mold was then baked in the oven at 65 °C for 4 h. The PDMS cell counter sheet was then placed on a glass slide and sealed to the glass slide with an oxygen plasma bonder for 2 min under a pressure of 80 W.

### Cell preparation

Cancer cell line A-549 was counted in the current experiment. A-549 cells were cultured in Dulbecco’s Modified Eagle’s medium (DMEM) supplemented with 10% Fetal Bovine Serum (FBS) and 1% Anti-Anti (Antibiotic–Antimycotic (100x)) and incubated at 37 °C in 5% CO_2_. For harvest, cells were rinsed with 0.25% Trypsin/0.53 mM EDTA to detach the cell layers. Trypsin (2 ml) was added, and the cells were placed in an incubator for 3 min. Cells were then washed with phosphate-buffered saline (PBS) and centrifuged. PBS was removed and 1 ml of DMEM was added. The number of cells in the suspension was determined by manual counting using a standard hemacytometer. Cell samples were diluted in a 9:1 ratio (PBS: cells suspension) to almost the target cell concentration range, such as 10^6^, 10^5^, 10^4^, 5 × 10^3^, 10^3^ cells/ml.

### Cell concentration measurement

For the multi-volume hemacytometer two cell concentration calculation models are needed. The Fixed-Volume Cell Counting (FVCC) model counted cells in a fixed volume of 0.1 µl which was repeated five times. The FVCC model is the conventional method used in a hemacytometer. The Multi-Volume Cell Counting (MVCC) model counted cells in multiple different volumes at once by calculating cells over an increasing slope per volume to measuring cell concentration. A 10 µl sample containing cells were loaded in the hemacytometer to standardize samples. After direct counting using a microscope with 5 squares (4 corners and the center squares), we calculated the mean and standard deviation of cell concentrations. The number of cells is divided by the chamber’s volume of 0.1 µl using the following formula:

1 $${\text{concentration}}~\left( {\text{{cells/ml}}} \right) = \frac{{{\text{Number~of~cells~}}}}{{0.1~\upmu {\text{l}}}}$$

Cell suspension of 35 µl were loaded in chip and counted after 5 min. For 5 min, most of the cells descend to the bottom of the chamber. So, all cells were counted from the bottom of the chip with a microscope focused on the bottom. A microscope with a 40 × objective capture 5 images of different areas in each chamber without changing focus. Each captured image has an area of 1 mm^2^. In the current experiment, we designed the four chambers to have heights of 0.1, 0.2, 0.4, and 0.8 mm. The equation used to calculate cell concentration is below.

2 $${\text{Chamber 1 }}\left( {{\text{volume}}{:}{\text{ }}0.{\text{1}}~\upmu {\text{l}}} \right){:}{\text{ concentration}}~\left( {\text{{cells/ml}}} \right) = \frac{{{\text{average~of~counted~cells~}} \times 1000{\text{~}}}}{{0.1~\upmu {\text{l}}}}$$

3 $${\text{Chamber 2 (volume{:}}}{\text{ 0.2 }}\upmu {\text{l}}){:}{\text{ concentration}}~\left( {\text{{cells/ml}}} \right) = \frac{{{\text{average~of~counted~cells~}} \times 1000{\text{~}}}}{{0.2~\upmu {\text{l}}}}$$

4 $${\text{Chamber 3 }}\left( {{\text{volume}}{:}{\text{ 0.4}}~\upmu {\text{l}}} \right){:}{\text{ concentration}}~\left( {\text{{cells/ml}}} \right) = \frac{{{\text{average~of~counted~cells~}} \times 1000{\text{~}}}}{{0.4~\upmu {\text{l}}}}$$

5 $${\text{Chamber 4 }}\left( {{\text{volume}}{:}{\text{ 0.8}}~\upmu {\text{l}}} \right){:}{\text{ concentration}}~\left( {\text{{cells/ml}}} \right) = \frac{{{\text{average~of~counted~cells~}} \times 1000{\text{~}}}}{{0.8~\upmu {\text{l}}}}$$

Cell concentrations were calculated by taking five images in a chamber. According to the sample cell concentrations, chambers among the four were selected to measure the cell concentration. If the concentration was low, a larger volume chamber was needed. If the concentration was high, 0.1 µl would be enough to measure the cell concentration. However, there were too many cells to count in a 0.8 µl chamber. Therefore, the volume of the chamber was chosen according to the cell concentration.

In the proposed multi-chamber hemacytometer, the Multi-Volume Cell Counting model (MVCC model) was applied to cell concentration. The cell numbers in each chamber from four images were plotted according to chamber volume and the slope of a linear equation between counted cell numbers and volume in multi-chambers was calculated to determine the concentration. The slope indicates the number of cells per volume (ml) in the graph of cell number by volume. In the current experiment, we counted four pictures in each chamber to find the cell concentration. The multi-volume model is derived from calculating the slope of the linear equation y = mx; m = slope and setting the intercept (x, y) at 0 as the counted cell number starts at 0 cell/ml. (Fig. 1) In the FVCC model, the large volume chamber has a wide cell concentration measurement range (Table 2). However, there are too many cells in the high concentration cell sample to be counted. In the proposed MVCC model, the total cell count was being reduced by more than 50% while the cell concentration measurement range remained the same.

**Table 2:** Total cell counts from cell concentration measurements.

Measure-ment method concentration (cells/ml)	Hema-cytometer (volume: 0.1 ul *5 )	Multi-volume hemacytometer				
		FVCC model*				MVCC Model ** (volume: 0.1 ul + 0.2 ul + 0.4 ul + 0.8 ul)
		Chamber 1 (volume: 0.1 ul * 5)	Chamber 2 (volume: 0.2 ul * 5)	Chamber 3 (volume: 0.4 ul * 5)	Chamber 4 (volume: 0.8 ul * 5)	
10^6^	**500**	**500**	**1000**	2000	4000	**1500**
10^5^	**50**	**50**	**100**	**200**	**400**	**150**
10^4^	5	5	**10**	**20**	**40**	**15**
5 × 10^3^	2.5	2.5	5	**10**	**20**	**7.5**
10^3^	0.5	0.5	1	2	4	1.5

*FVCC Model: Fixed-Volume Cell Counting Model.

**MVCC Model: Multi-Volume Cell Counting Model.

Bold values Cell concentration measurement range.
